# Identification of a Major Determinant for Serine-Threonine Kinase Phosphoacceptor Specificity

**DOI:** 10.1016/j.molcel.2013.11.013

**Published:** 2014-01-09

**Authors:** Catherine Chen, Byung Hak Ha, Anastasia F. Thévenin, Hua Jane Lou, Rong Zhang, Kevin Y. Yip, Jeffrey R. Peterson, Mark Gerstein, Philip M. Kim, Panagis Filippakopoulos, Stefan Knapp, Titus J. Boggon, Benjamin E. Turk

**Affiliations:** 1 Department of Pharmacology, Yale University School of Medicine, New Haven, CT 06520, USA; 2 Department of Molecular Biophysics and Biochemistry, Yale University, New Haven, CT 06520, USA; 3 Department of Computer Science and Engineering, The Chinese University of Hong Kong, Shatin, New Territories, Hong Kong; 4 Division of Basic Science, Fox Chase Cancer Center, Philadelphia, PA 19111, USA; 5 Donnelly Centre for Cellular and Biomolecular Research, University of Toronto, Toronto, ON M5S 3E1, Canada; 6 Oxford University, Nuffield Department of Clinical Medicine, Target Discovery Institute (TDI) and Structural Genomics Consortium (SGC), Oxford OX3 7FZ, UK; 7 Ludwig Institute for Cancer Research, Old Road Campus Research Building, Oxford OX3 7DQ, UK

## Abstract

Eukaryotic protein kinases are generally classified as being either tyrosine or serine-threonine specific. Though not evident from inspection of their primary sequences, many serine-threonine kinases display a significant preference for serine or threonine as the phosphoacceptor residue. Here we show that a residue located in the kinase activation segment, which we term the “DFG+1” residue, acts as a major determinant for serine-threonine phosphorylation site specificity. Mutation of this residue was sufficient to switch the phosphorylation site preference for multiple kinases, including the serine-specific kinase PAK4 and the threonine-specific kinase MST4. Kinetic analysis of peptide substrate phosphorylation and crystal structures of PAK4-peptide complexes suggested that phosphoacceptor residue preference is not mediated by stronger binding of the favored substrate. Rather, favored kinase-phosphoacceptor combinations likely promote a conformation optimal for catalysis. Understanding the rules governing kinase phosphoacceptor preference allows kinases to be classified as serine or threonine specific based on their sequence.

## Highlights

• A single active site residue can determine kinase phosphoacceptor specificity
• Favored and disfavored substrates promote distinct kinase-bound conformations
• A simple rule predicts kinase phosphoacceptor preference from its DFG+1 residue

## Graphical Abstract

**Figure:**
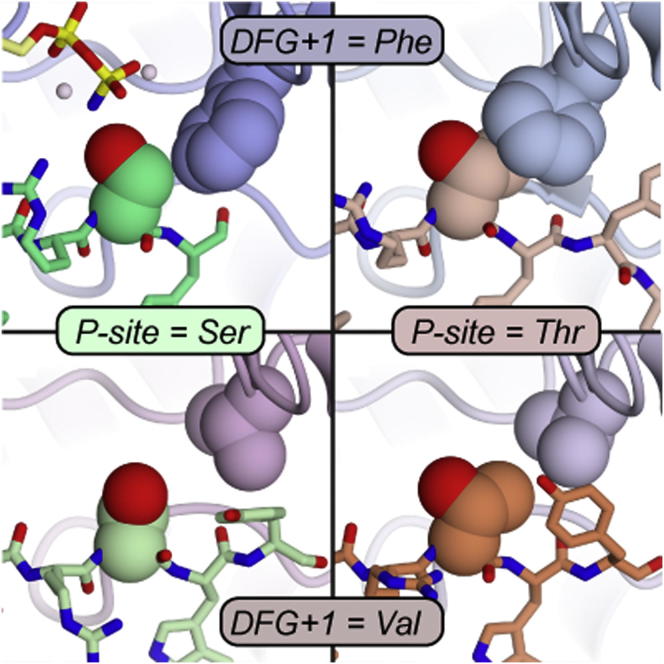

## Introduction

Proper signal transmission by protein kinases requires that they phosphorylate specific substrates at defined sites. Multiple mechanisms can act in concert to provide substrate specificity for kinases, including kinase-substrate colocalization, compartmentalization through the use of scaffold proteins, substrate recruitment through kinase adaptor subunits, and direct physical interactions between kinases and their substrates (Ubersax and Ferrell, 2007). In order for substrate phosphorylation to occur, the residue phosphorylated by the kinase must bind at least transiently to the catalytic cleft of the kinase. Accordingly, protein kinases tend to phosphorylate substrates in the context of consensus sequence motifs that have complementarity to the kinase active site (Pinna and Ruzzene, 1996). Such kinase phosphorylation site motifs play an important role in targeting kinases to specific substrates within the cell, as well as directing kinases to phosphorylate specific sites on their substrates.

One important aspect of the kinase phosphorylation site motif is the identity of the phosphorylation site residue itself. Almost all kinases in eukaryotes phosphorylate protein substrates on Ser, Thr, or Tyr residues. Ser-Thr kinases, which make up approximately 80% of the human kinome, comprise several groups phylogenetically distinct from Tyr-specific kinases (Manning et al., 2002). Accordingly, Ser-Thr kinases possess conserved signature residues within the catalytic domain important for accommodating a small, aliphatic phosphoacceptor residue at the active site (Taylor et al., 1995). Likewise, a distinct set of conserved residues characterizes Tyr kinases, which must accommodate a large, aromatic residue. Interestingly many, though not all, Ser-Thr kinases are significantly selective for either Ser or Thr as the phosphoacceptor residue (Pinna and Ruzzene, 1996). For example, cAMP-dependent protein kinase (PKA) strongly favors Ser over Thr in peptide substrates, and a large majority (>90%) of established in vivo phosphorylation sites are at Ser residues (Aimes et al., 2000, Kemp et al., 1977, Shabb, 2001). Conversely, the kinase LKB1 activates numerous downstream protein kinase substrates by phosphorylation exclusively on Thr residues (Lizcano et al., 2004). Although substrate specificity studies have predominantly been conducted in vitro, evidence is now emerging indicating that phosphoacceptor residue identity significantly influences substrate phosphorylation efficiency in living cells (Kang et al., 2013). Thus, the presence of a preferred phosphoacceptor residue appears to be important for targeting of specific substrates by Ser-Thr kinases. However, the rules that govern this specificity are yet to be defined.

Here, we show that phosphoacceptor preference of Ser-Thr kinases is determined largely by the identity of a single residue, which we term “DFG+1.” This residue is located within the kinase activation segment, a conformationally flexible loop important for kinase regulation. Mutagenesis of this residue is sufficient to change the phosphoacceptor preference for kinases from distinct groups in a predictable manner. Identification of this residue therefore establishes a simple rule that can be used to predict phosphorylation site preference for a kinase of unknown specificity. We go on to show by X-ray crystallography of kinase-peptide complexes that conformation, rather than binding affinity, drives phosphoacceptor preference. Overall, these studies explain how a protein kinase is able to discriminate between Ser and Thr, two residues that differ only by a single methyl group.

## Results

### A Residue within the Kinase Activation Loop Covaries with Phosphorylation Site Preference

We recently analyzed the peptide substrate specificity of a large number of Ser-Thr kinases from *S. cerevisiae* (Mok et al., 2010). Among these kinases, 32 preferred Ser, 10 preferred Thr, and 14 lacked substantial phosphoacceptor residue preference (Table S1 available online). While about half of the nonselective kinases belonged to a single phylogenetic group (the CDK-, MAPK-, GSK3-, and CKII-related [CMGC] kinase group), possibly attributable to a distinct peptide-binding mode (Brinkworth et al., 2003, Brown et al., 1999, Mok et al., 2010), all other kinase groups included both Ser- and Thr-specific kinases (Table S1 and Figure S1). Thus, in contrast to the clear distinction between Tyr kinases and Ser-Thr kinases, Ser-preferring and Thr-preferring kinases cannot be readily distinguished based on phylogeny.

Residues within the kinase catalytic domain that determine target specificity covary with corresponding residues in the substrate peptide (Li et al., 2003). Computational analysis of our yeast kinase data identified residues that covaried between kinases and their consensus peptide sequences (Yip et al., 2011). One residue correlating strongly with phosphoacceptor preference is found immediately downstream of a conserved Asp-Phe-Gly (DFG) sequence at the N terminus of the kinase activation loop (hereafter referred to as the DFG+1 residue). Consistent with a potential role in controlling kinase specificity, this residue is located within the substrate-binding cleft (Goldsmith et al., 2007). Among yeast kinases analyzed, Ser-selective kinases had larger hydrophobic residues (predominantly Leu, Phe, and Met) at this position, while most Thr-specific kinases had the β-branched aliphatic residue Ile at this position. All nonselective kinases had either Leu or Ser as the DFG+1 residue. This correlation was extended by inspection of additional human kinases (Table S2). For example, LKB1 and PDK1, which phosphorylate primarily Thr residues in substrates (Lizcano et al., 2004, Mora et al., 2004), have the β-branched residues Val and Thr, respectively, at the DFG+1 position. In addition, human protein kinase C isozymes, which prefer Ser as the phosphoacceptor (Ferrari et al., 1985, House et al., 1987), have the straight-chain aliphatic residue Met at the analogous position. Based on its location within the kinase fold and the strong correlation between its identity and kinase specificity, we hypothesized that the DFG+1 residue might be an important determinant of phosphoacceptor specificity for Ser-Thr kinases.

### The DFG+1 Residue Controls Phosphoacceptor Specificity of Multiple Ser-Thr Kinase Families

**Figure 1:**
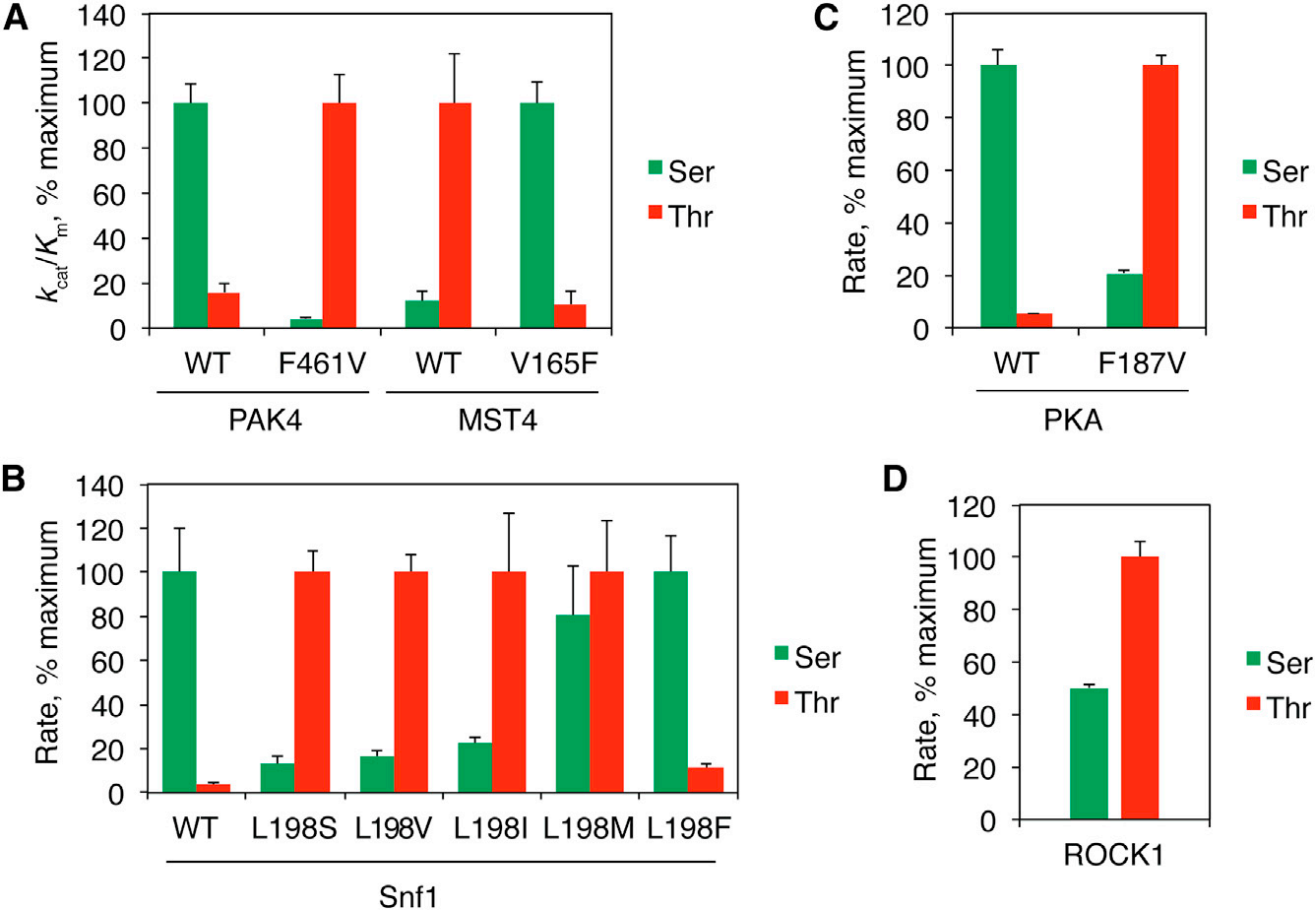
The DFG+1 Residue Controls Ser-Thr Phosphosite Specificity For the kinases and peptide substrates indicated, the relative phosphorylation rates, as determined by radiolabel incorporation assay, are shown as a percentage of the maximally active substrate. The phosphorylation site residue is indicated by the color and location of the bar (Ser, green/left; Thr, red/right). (A) PAK4 and MST4 DFG+1 mutants exchange phosphorylation site specificity. Relative *k*_cat_/*K*_m_ values for WT PAK4, PAK4^F461V^, WT MST4, and MST4^V165F^ were derived from data shown in Table 1. (B) Relative reaction rates for phosphorylation of peptides with the sequence ALARAA(**X**)AAALAKKK at 40 μM, where X is Ser or Thr as indicated, by yeast Snf1 and the indicated mutants. Results are the mean ±SEM from three separate determinations. (C) Relative rates of WT PKA and PKA^F187V^ phosphorylation of 10 μM peptide (GGRRRRR(**X**)WYFGGGK). Results show mean ±SEM of triplicate samples from a representative experiment. (D) Relative rates of phosphorylation of the peptides ARKRERAY(**X**)FGHHA at 5 μM with the indicated phosphoacceptor residue by ROCK1. Results show mean ±SEM of duplicate samples from a representative experiment. Absolute reaction rates for (B)–(D) are provided in Table S3. See also Figure S1 and Tables S1 and S2.

Based on this hypothesis, mutation of the DFG+1 residue within Ser-Thr kinases should alter specificity in a predictable manner. We initially focused on human kinases in the Ste7-, Ste11-, and Ste20-related (STE) group. Most kinases in this group, including those in the mammalian Ste20-like (MST) kinase family, are Thr selective (Miller et al., 2008), while members of the p21-activated kinase (PAK) family are Ser selective (Rennefahrt et al., 2007). Phosphorylation site specificity within these families correlates with DFG+1 residue identity (Val for MST kinases and Phe for PAKs). We generated DFG+1 exchange mutants of these kinases (MST4^V165F^ and PAK4^F461V^) and examined their phosphorylation specificity alongside their wild-type (WT) counterparts using optimized synthetic peptides that differ only at the phosphosite. As anticipated, WT MST4 preferentially phosphorylated the Thr peptide, while WT PAK4 preferentially phosphorylated the Ser peptide (Figure 1A and Table 1). Furthermore, mutation of the DFG+1 residue was sufficient to invert phosphorylation site specificity for both kinases on both consensus peptide substrates and peptide libraries (Figures 1A and S2). Notably, we found that for a given kinase, the *K*_m_ values for phosphorylation of the Ser and Thr peptides were similar, but phosphoacceptor residue identity had a large effect on *k*_cat_ (Table 1). These results suggest that phosphoacceptor preference is not mediated by differences in substrate binding affinity.

**Table 1:** Catalytic Constants for Phosphorylation of Peptide Substrates by PAK4 and MST4 and Their Exchange Mutants

Kinase	Phosphoacceptor Residue	*k*_cat_ (s^−1^)	*K*_m_ (μM)	*k*_cat_*/K*_m_ (M^−1^s^−1^)
WT PAK4	Ser	0.097 ± 0.015	280 ± 20	340 ± 30
	Thr	0.0089 ± 0.0009	170 ± 20	54 ± 12
PAK4^F461V^	Ser	0.0022 ± 0.0001	129 ± 3	16.8 ± 0.5
	Thr	0.0301 ± 0.0003	75 ± 1	400 ± 50
WT MST4	Ser	0.6 ± 0.2	21.7 ± 0.7	25,000 ± 9,970
	Thr	3.1 ± 0.9	14 ± 1	210,000 ± 46,000
MST4^V165F^	Ser	0.11 ± 0.02	360 ± 30	310 ± 30
	Thr	0.013 ± 0.002	390 ± 90	33 ± 2

Substrate peptides had the sequence GGRRRRR**X**WYFGGGK for PAK4 and NKGYN**X**LRRKK for MST4, where **X** was either Ser or Thr, as indicated above. Data show the mean of two separate experiments ±SEM. See also Figure S2.

To determine whether the DFG+1 residue controls phosphoacceptor preferences among Ser-Thr kinases in general, we tested the effect of mutating this residue in two more distantly related kinases: Snf1 (the yeast ortholog of AMP-activated protein kinase, from the Ca^2+^/calmodulin-dependent protein kinase-related group) and PKA (from the PKA-, PKG-, and PKC-related [AGC] group). We generated a series of Snf1 mutants in which the DFG+1 residue (Leu198) was changed to residues most commonly found in other kinases at this position and evaluated the ability of these mutants to phosphorylate Ser- and Thr-containing peptides (Figure 1B). Mutation of Leu198 to a small or β-branched residue (Ser, Val, or Ile) converted it to a Thr kinase, while mutation to Phe in Snf1^L198F^ retained a preference for Ser. Unexpectedly, Snf1^L198M^, predicted to be Ser specific, did not discriminate between Ser and Thr. For PKA, mutation of the DFG+1 residue (Phe187) to Val inverted its selectivity from Ser to Thr specific (Figure 1C). Taken together, these data indicate that the DFG+1 residue plays a predominant role in dictating phosphoacceptor specificity across multiple kinase groups.

If the DFG+1 residue is the primary determinant of Ser-Thr kinase phosphoacceptor specificity, the identity of this residue should allow us to predict the preference of an uncharacterized kinase. Most kinases within the human AGC group appear to prefer Ser, and in keeping with this, Leu, Met, and Phe are found most frequently at the DFG+1 position. Kinases in the ROCK family are outliers in that they have a β-branched Thr DFG+1 residue, suggesting they are likely to prefer Thr over Ser. Using a pair of matched peptides differing only at the phosphoacceptor site, we found that ROCK1 indeed had a significant (approximately 2-fold) preference for Thr as the phosphoacceptor (Figure 1D).

**Figure 2:**
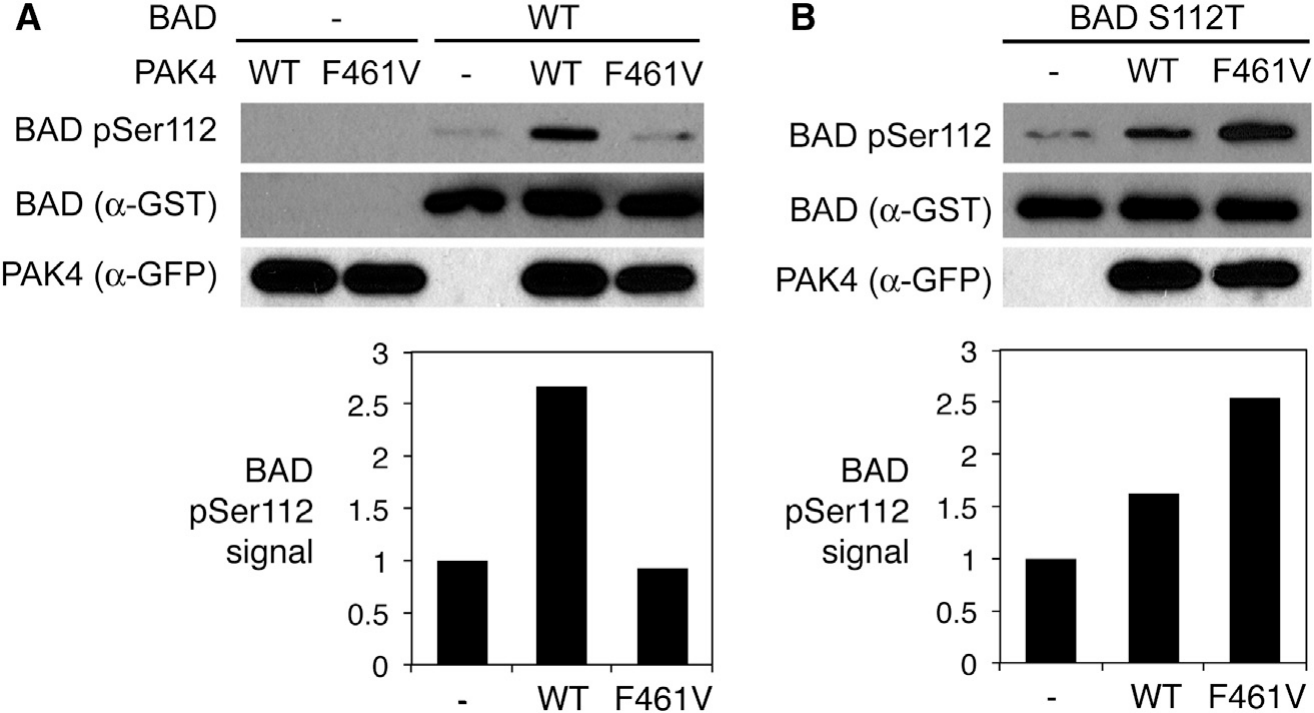
The DFG+1 Residue Controls Protein Phosphorylation in Cells (A) GST-tagged BAD was transiently expressed in HEK293 cells with GFP-tagged WT PAK4 or PAK4^F461V^ catalytic domain. Following serum starvation and treatment with wortmannin to reduce phosphorylation by endogenous kinases, GST-BAD was purified from cell lysates and analyzed by immunoblotting using phosphospecific antibodies. Quantified signal intensities for BAD pSer112 normalized to the total BAD signal are shown below as the ratio to the background signal. (B) Phosphorylation of GST-tagged BAD^S112T^ following coexpression with WT PAK4 and PAK4^F461V^ was analyzed as in (A). A 5-fold greater quantity of BAD^S112T^ compared to WT BAD was loaded on the gel to compensate for the reduced reactivity of the BAD pSer112 antibody.

To assess the contribution of the DFG+1 residue to substrate phosphorylation in cells, we examined the ability of PAK4 to phosphorylate an established substrate, the proapoptotic protein BAD (Gnesutta et al., 2001), upon mutation of either PAK4 or its phosphorylation site in BAD (Ser112 to Thr, resulting in BAD^S112T^). Coexpression of WT PAK4 catalytic domain with BAD in HEK293 cells yields robust phosphorylation at Ser112 (Figure 2). Despite having similar catalytic parameters to the WT kinase in vitro on peptide substrates (see Table 1), PAK4^F461V^ expression did not detectably induce WT BAD phosphorylation in cells above the background level. In contrast, PAK4^F461V^ could phosphorylate BAD^S112T^ in cells, but WT PAK4 phosphorylation of BAD^S112T^ was substantially reduced. These results suggest that an optimal combination of phosphorylation site residue and DFG+1 residue is essential for maximal phosphorylation of at least some kinase substrates in living cells.

### The Structural Basis for Ser-Thr Kinase Phosphoacceptor Specificity

**Figure 3:**
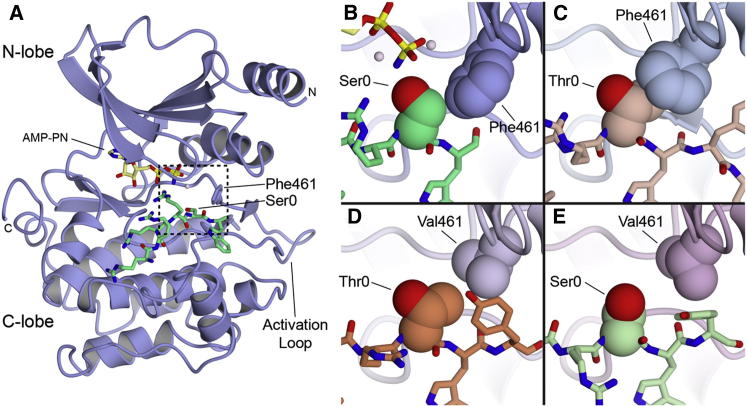
Structural Analysis of PAK4 and PAK4^f461v^ Complexes with PAKtide-S and PAKtide-T (A) Overall structure of PAK4 in complex with a PAKtide peptide. WT PAK4 with PAKtide-S is shown, with PAK4 in cartoon format and PAKtide-S in stick format. PAK4 is colored blue and PAKtide-S is colored green. DFG+1 residue Phe461 is indicated and shown in stick format. Phosphoacceptor residue Ser0 is indicated. Nucleotide is shown in stick format. Kinase N- and C-lobes are indicated. A box indicates the region shown in (B)–(E). (B–E) Close-ups of (B) PAK4 WT with PAKtide-S, (C) PAK4 WT with PAKtide-T, (D) PAK4^F461V^ with PAKtide-T, and (E) PAK4^F461V^ with PAKtide-S are shown. The phosphoacceptor residue Ser0 or Thr0 and the DFG+1 residue Phe461 or Val461 are shown as spheres. Structural figures were generated using CCP4mg (McNicholas et al., 2011). See also Figure S3.

To understand the structural basis for how the DFG+1 residue controls phosphoacceptor specificity, we determined the X-ray cocrystal structures of WT and F461V mutant PAK4 catalytic domains in complex with consensus peptide substrates incorporating both their favored and disfavored phosphorylation site residues (PAKtide-S and PAKtide-T, Figure 3). We also determined an additional X-ray cocrystal structure of WT PAK4 with a longer version of the consensus peptide, PAKtide-S(L) (Figure S3). As previously observed for WT PAK4 in the absence of bound peptide (Eswaran et al., 2007, Ha et al., 2012), in each of the structures PAK4 is observed in the active state conformation with the activation loop phosphorylated on residue Ser474. The maximal root-mean-square deviation between the five structures is 1.1 Å over 290 C-α atoms (Table 2). Interestingly, the cocrystallized ATP analog was only visible in the electron density for the structure of the WT PAK4 complex with PAKtide-S (Figure S3). Both structures of WT PAK4 in complex with the optimal substrate, PAKtide-S or PAKtide-S(L), are highly similar, with small differences observed in the conformation of several side chains within the bound peptide and some crystal packing-induced conformational differences C-terminal to the phosphoacceptor residue (Figure S3). The bound peptide has a nearly identical backbone conformation, and, importantly, the residues most strongly selected by the kinase (the P-2 Arg and the P0 Ser residues [Rennefahrt et al., 2007, Zhu et al., 2005]) are observed in very similar orientations. As found in other structures of Ser-Thr kinases with bound peptide substrates (Brown et al., 1999, Bullock et al., 2005, Madhusudan et al., 1994, Yang et al., 2002), in both structures the Ser hydroxyl points toward the ATP binding site and is engaged in a network of polar contacts with conserved residues in the kinase catalytic loop (Figures 3A and S3L). Both structures therefore show the PAK4-peptide complex in a conformation apparently competent for phosphate transfer to occur. The DFG+1 Phe residue in these WT PAK4 structures is positioned within 4Å of the Ser phosphoacceptor β-carbon and oriented perpendicular to the substrate peptide backbone (Figure 3B). This conformation for the DFG+1 Phe would preclude the additional methyl group of a Thr phosphoacceptor due to steric hindrance. Indeed, in the structure of WT PAK4 in complex with PAKtide-T, the bound peptide is in a nearly identical conformation. However, to accommodate the Thr phosphoacceptor residue, the DFG+1 Phe undergoes significant conformational movement: it rotates ∼75° about its β-γ bond to allow the face of the Phe aromatic ring to interact with the Thr methyl group (Figure 3C). This conformation for the DFG+1 residue likely interferes with positioning of the γ-phosphate of ATP (Figures S3M–S3O). Consistent with the kinetic data, the structure does not suggest impaired PAKtide-T binding to the WT kinase. Rather, decreased phosphorylation of Thr substrates may occur due to conformational effects within the catalytic center. Taken together, these structures provide a likely explanation for why WT PAK4, and by extension other kinases with a DFG+1 Phe residue, is selective for Ser at the phosphorylation site.

**Table 2:** Data Collection and Refinement Statistics

	PAK4 + PAKtide-T	PAK4 + PAKtide-S	PAK4^F461V^ + PAKtide-T	PAK4^F461V^ + PAKtide-S	PAK4 + PAKtide-S(L)
	PDB ID: 4JDH	PDB ID: 4JDI	PDB ID: 4JDJ	PDB ID: 4JDK	PDB ID: 2Q0N
**Data Collection**					

Space group	*P*4_1_2_1_2	*P*4_1_2_1_2	*P*4_1_2_1_2	*P*4_1_2_1_2	*P*4_3_2_1_2
X-ray source and detector	NSLS X25 ADSC Q315	APS 24-ID-E ADSC Q315	NSLS X25 ADSC Q315	NSLS X25 ADSC Q315	SLS X10SA Mar-225
Wavelength (Å)	1.10000	0.97922	1.10000	1.10000	0.97910
Unit cell: a, b, c (Å)	61.9, 61.9, 179.4	61.9, 61.9, 181.6	61.8, 61.8, 181.7	61.6, 61.6, 180.8	145.7, 145.7, 39.6
α, β, γ (°)	90, 90, 90	90, 90, 90	90, 90, 90	90, 90, 90	90, 90, 90
Resolution range (Å)^a^	50.0–2.0 (2.07–2.0)	30.0–1.85 (1.92–1.85)	50.0–2.3 (2.38–2.3)	50.0–2.4 (2.49–2.4)	50.0–1.75 (1.81–1.75)
Number of unique reflections	24,692	31,180	30,247	14,691	40,410
Completeness^a^ (%)	100 (100)	99.9 (100)	100 (99.8)	100 (100)	92.5 (94.2)
*R*_sym_ (%)^a^	7.0 (119.0)	9.1 (159.6)	8.9 (69.0)	13.3 (96.4)	5.7 (35.5)
*R*_pim_ (%)^a^	2.2 (34.9)	3.0 (52.2)	4.0 (36.7)	3.8 (25.0)	–
*R*_rim_ (%)^a^	7.7 (124.1)	9.2 (93.4)	9.7 (79.4)	12.8 (81.7)	–
Mean σ^a^	27.9 (1.7)	22.8 (1.8)	17.5 (2.2)	17.4 (1.9)	16.3 (4.3)
Redundancy	12.4 (12.8)	10.3 (10.5)	6.0 (5.0)	11.9 (12.0)	12.0 (5.6)

**Refinement Statistics**					

Resolution range (Å)^a^	50.0–2.0 (2.05–2.0)	30.0–1.85 (1.9–1.85)	50.0–2.3 (2.36–2.3)	50–2.4 (2.46–2.4)	32.6–1.75 (1.79–1.75)
*R*_factor_ (%)^a^	18.3 (22.2)	19.2 (26.6)	19.5 (27.3)	18.5 (26.2)	18.2 (24.4)
Free *R*_factor_ (%)^a^	22.4 (27.2)	23.3 (27.2)	24.4 (29.4)	24.9 (31.0)	22.8 (33.2)
Free *R* reflections (%)^a^	5.1 (5.6)	5.1 (5.1)	5.1 (8.0)	5.0 (6.8)	5.0 (5.7)
Free *R* reflections, number^a^	1,250 (70)	1,568 (80)	835 (67)	725 (49)	2,020 (159)
Residues built					
PAK4	A/300–589	A/300–589	A/300–589	A/300–589	A/291–591
Cocrystallized peptide	B/−3 to 3	B/−4 to 1	B/−3 to 2	B/−3 to 2	B/−5 to 5
Number water molecules	70	139	102	49	251
Mean *B* factor (Å^2^)					
Protein (A)	45.5	35.5	39.3	48.0	24.0
AMP-PN (A)	–	30.9	–	–	–
Mg^2+^ (A)	–	34.5	–	–	–
Peptide (B)	66.1	47.7	75.0	85.1	26.3
H_2_0	47.0	40.3	39.2	46.5	34.1

**Model Statistics**					

rmsd bond lengths (Å)	0.019	0.008	0.007	0.014	0.016
rmsd bond angles (°)	2.007	1.328	1.179	1.803	1.588
Ramachandran plot (%) favored/allowed/disallowed	98.3/1.7/0	98.0/2.0/0	97.9/2.1/0	97.2/2.8/0	98.1/1.9/0

a High-resolution shell.

To understand why kinases with a β-branched DFG+1 residue favor Thr, we examined the cocrystal structures of PAK4^F461V^ in complex with PAKtide-S and with PAKtide-T. In the cocrystal structure of PAK4^F461V^ with PAKtide-T, the phosphoacceptor Thr methyl group stacks snugly against the mutated DFG+1 Val residue, positioning the phosphoacceptor hydroxyl in an orientation similar to that observed in the WT PAK4-PAKtide-S complex (Figure 3D). The phosphoacceptor Thr appears poised for phosphate transfer, and thus the combination of Val at the DFG+1 position and Thr at the phosphoacceptor site seems to be highly accommodative to positioning an active-like conformation for the phosphoacceptor hydroxyl. In striking contrast, the cocrystal structure of PAK4^F461V^ in complex with PAKtide-S displays an unexpected conformation of the phosphoacceptor Ser residue (Figure 3E). Although the overall peptide conformation is similar, the phosphoacceptor Ser is rotated about its α-β bond (χ_1_) such that the hydroxyl group is pointed away from the active site. Additionally, two alternate conformations are observed for the DFG+1 Val residue. The PAK4^F461V^ cocrystal structures therefore suggest that Val at the DFG+1 residue favors Thr over Ser because the additional methyl group allows interplay between the DFG+1 and phosphoacceptor residues, correctly positioning them to promote an optimal conformation for phosphate transfer.

## Discussion

Our results suggest that discrimination by kinases between Ser and Thr involves control of proper conformation rather than binding to the active site. Our conclusion that Ser and Thr peptides bind with equal affinity is partly based on our observation that PAK4 and MST4 phosphorylate Ser and Thr substrates with similar *K*_m_ values. However, because the *K*_m_ value for an enzyme reaction is not necessarily equivalent to the substrate dissociation constant, substrate binding, conformation, and product release could all theoretically contribute to phosphoacceptor discrimination. We note, however, that Ser and Thr peptides appear to bind with equal affinity to the Ser-selective kinase PKA (Aimes et al., 2000). Discrimination between Tyr and Ser/Thr by kinases appears to involve differences in binding affinity as well as conformational control. X-ray crystal structures of Tyr kinases in complex with substrates have revealed favorable nonpolar interactions between the phosphoacceptor Tyr and conserved Tyr kinase-specific residues within the active site cleft (Favelyukis et al., 2001, Hubbard, 1997). In addition, Tyr kinases and Ser-Thr kinases differ in the conformation of the so-called P+1 loop, which interacts with the peptide backbone of a bound substrate to control its distance from the active site, thus dictating the size of the phosphoacceptor that can be accommodated (Hubbard, 1997, Taylor et al., 1995). Comparative kinetic analyses have been conducted for Ser and Tyr peptide phosphorylation by casein kinase 2 (CK2), a kinase that belongs to the CMGC Ser-Thr kinase group but that can also phosphorylate on Tyr residues, albeit with greatly reduced catalytic efficiency. These analyses revealed large (100-fold or more) differences in both *k*_cat_ and *K*_m_, as well as distinct sequence preferences at residues flanking the phosphoacceptor (Marin et al., 1999, Vilk et al., 2008). These results suggest that Ser and Tyr peptides bind in distinct modes to the active site of CK2 and imply different binding affinities. In contrast, we found that Ser and Thr peptides bound to PAK4 with an identical backbone conformation and apparently comparable affinities.

The preference of a kinase for Ser or Thr, as reflected in peptide substrate phosphorylation in vitro, is likely to be generally important for substrate targeting in vivo. For example, we observed that most kinases in yeast prefer Ser, and based on the identity of their DFG+1 residues, most human kinases are predicted to prefer Ser as well. These observations suggest that the preponderance of pSer among yeast and human phosphoproteomes (Hornbeck et al., 2012, Sadowski et al., 2013) is related to intrinsic biochemical properties of eukaryotic kinases. For some kinases the presence of the preferred phosphoacceptor residue may constitute an essential component of its phosphorylation site motif. However, in many cases the preferred phosphoacceptor, while not absolutely required for phosphorylation by the kinase, may serve to confer robustness on substrate phosphorylation. For example, recent work has shown that exchanging Thr for Ser at sites of phosphorylation by the mTOR kinase could modulate sensitivity to activating stimuli or inhibitors of the kinase (Kang et al., 2013). In addition to influencing recognition by kinases, phosphorylation site identity can also influence downstream signaling as both protein phosphatases and phosphoprotein interaction domains can discriminate between pSer and pThr (Bremmer et al., 2012, Durocher et al., 2000).

While we have identified the DFG+1 residue as a major determinant for several kinases, other residues within the catalytic domain undoubtedly contribute to phosphoacceptor preference as well. Individual kinases vary widely with respect to the stringency with which they discriminate between the two residues, and this is likely to be controlled by additional active site residues. For example, a residue in the glycine-rich loop located proximal to the γ-phosphate of bound ATP was shown to influence the extent to which PKA prefers Ser (Aimes et al., 2000). In addition, we found that a DFG+1 Leu residue correlated with Ser preference for most kinases, yet for kinases in the CMGC group it was associated with a lack of phosphoacceptor selectivity. Identification of additional “modifier” residues will facilitate a more complete understanding of structural features controlling kinase specificity. In addition, for some kinases it is possible that phosphorylation site specificity is entirely determined by other residues. Nonetheless, our current study suggests that, by inspection, one may determine whether a kinase is selective for Ser or Thr. To wit, a β-branched residue at the DFG+1 position appears to invariably dictate a preference for Thr, but Phe dictates preference for Ser as the phosphoacceptor residue. Our study therefore provides a much improved understanding of how protein kinases discriminate between phosphorylation site residues.

## Experimental Procedures

### Protein Expression and Purification

PAK4, MST4, and PKA (WT and point mutants) were expressed with N-terminal hexahistidine tags in *E. coli* and purified by immobilized metal affinity chromatography. PAK4 and MST4 were further subjected to anion exchange, followed by size exclusion chromatography or size exclusion chromatography alone, respectively. GST-Snf1 (WT and mutant) was expressed in *E. coli*, purified by one-step glutathione affinity chromatography, and then activated by phosphorylation with recombinant Elm1 kinase, as described (Lee et al., 2012). Descriptions of expression vectors used and detailed purification procedures are provided in Supplemental Experimental Procedures.

### Protein Kinase Assays

Kinase activity toward peptide substrates was determined by filter binding assay using radiolabeled ATP. Kinases were incubated with peptides and [γ-^33^P]ATP at 30°C for varying times, and aliquots were spotted on to P81 filter discs. Filters were quenched in 75 mM phosphoric acid and then washed three times for 4 min in 75 mM phosphoric acid, washed once briefly in acetone, air-dried, and analyzed by liquid scintillation counting. The amount of product formed was calculated from a standard curve made using [γ-^33^P]ATP at the same specific activity. Initial rates were determined by fitting the data to a line with Excel, and catalytic constants were calculated by fitting the initial velocity data to the Michaelis-Menten equation using KaleidaGraph software. Detailed procedures, including buffer compositions for each kinase reaction, are provided in Supplemental Experimental Procedures.

### Cell Culture and Transfection

HEK293 cells were cultured at 37°C and 5% CO_2_ in Dulbecco’s modified Eagle’s medium containing 4.5 g/l D-glucose and L-glutamine (Invitrogen), 10% fetal bovine serum (FBS), and 1% penicillin/streptomycin. Cells in 6-well plates were cotransfected with 0.4 μg pEGFP-PAK4 catalytic domain and 3.6 μg pEBG-BAD using Lipofectamine 2000 (Invitrogen). After 24 hr, cells were exchanged into reduced serum medium (0.1% FBS) for 16 hr and treated with 250 nM wortmannin for 30 min prior to lysis. Cell lysates were prepared by washing briefly with PBS and extracting into lysis buffer (20 mM Tris-HCl [pH 7.5], 150 mM NaCl, 1 mM EDTA, 1 mM EGTA, 1% Triton X-100, 2.5 mM sodium pyrophosphate, 1 mM β-glycerophosphate, 1 mM Na3VO4, 1 mM DTT, 1 mM PMSF, 10 μg/ml leupeptin, 2 μg/ml pepstatin, and 10 μg/ml aprotinin) for 10 min at 4°C with rotation and centrifuging (10 min at 13,500 × *g*). GST-tagged BAD was purified from the supernatant with 20 μl glutathione Sepharose 4B suspension (GE Healthcare). Samples were subjected to SDS-PAGE followed by immunoblotting with α-BAD pSer112 antibody (Cell Signaling Technology #9291), α-GST antibody (Cell Signaling Technology #2624) and α-GFP antibody (Clontech #632381), and detection by enhanced chemiluminescence.

### Crystallography

Crystals of WT PAK4 and PAK4^F461V^ catalytic domain were obtained with both PAKtide-T (GGRRRRRTWYFGGGK) and PAKtide-S (RRRRSWY) using the vapor diffusion hanging drop method. For WT PAK4 with peptides, we grew WT PAK4 crystals against 0.1 M Tris-HCl (pH 7.5) and 1.5–2.0 M NaOAc at room temperature and soaked with peptides. For PAK4^F461V^ cocrystals with peptide, we grew against conditions of 0.1 M Tris-HCl (pH 7.5) and 1.5–2.0 M NaOAc at room temperature. For cocrystals of PAK4 in complex with PAKtide-S(L) (RRRRRSWYFDG), we grew against 1.7 M ammonium sulfate, 15% PEG400, and 0.1 M Tris-HCl (pH 8.0). Crystallographic data were collected at Advanced Photon Source (APS) beamline 24-ID-E, National Synchrotron Light Source (NSLS) beamline X25, or the Swiss light source beamline SLS-X10. Following structure solution, good electron density was observed for each of the kinase domains and their bound peptides.

## Accession Numbers

Crystallographic coordinates and structure factors are deposited in the PDB under accession codes 4JDH, 4JDI, 4JDJ, 4JDK, and 2Q0N.

## Supplemental Information

Document S1. Figures S1–S3, Tables S1–S3, and Supplemental Experimental Procedures
