# Supplementary material for: Identification of a Major Determinant for Serine-Threonine Kinase Phosphoacceptor Specificity
Source: Mol Cell. 2014 Jan 9;53(1):140–7. doi: 10.1016/j.molcel.2013.11.013 (PMC3898841; doi:10.1016/j.molcel.2013.11.013)
Supplement: Document S1. Figures S1–S3, Tables S1–S3, and Supplemental Experimental Procedures [file mmc1.pdf]

Molecular Cell, Volume *53*

Supplemental Information

Identification of a Major Determinant

for Serine-Threonine Kinase

Phosphoacceptor Specificity

Catherine Chen, Byung Hak Ha, Anastasia F. Thévenin, Hua Jane Lou, Rong Zhang, Kevin Y. Yip, Jeffrey R. Peterson, Mark Gerstein, Philip M. Kim, Panagis Filippakopoulos, Stefan Knapp, Titus J. Boggon, and Benjamin E. Turk

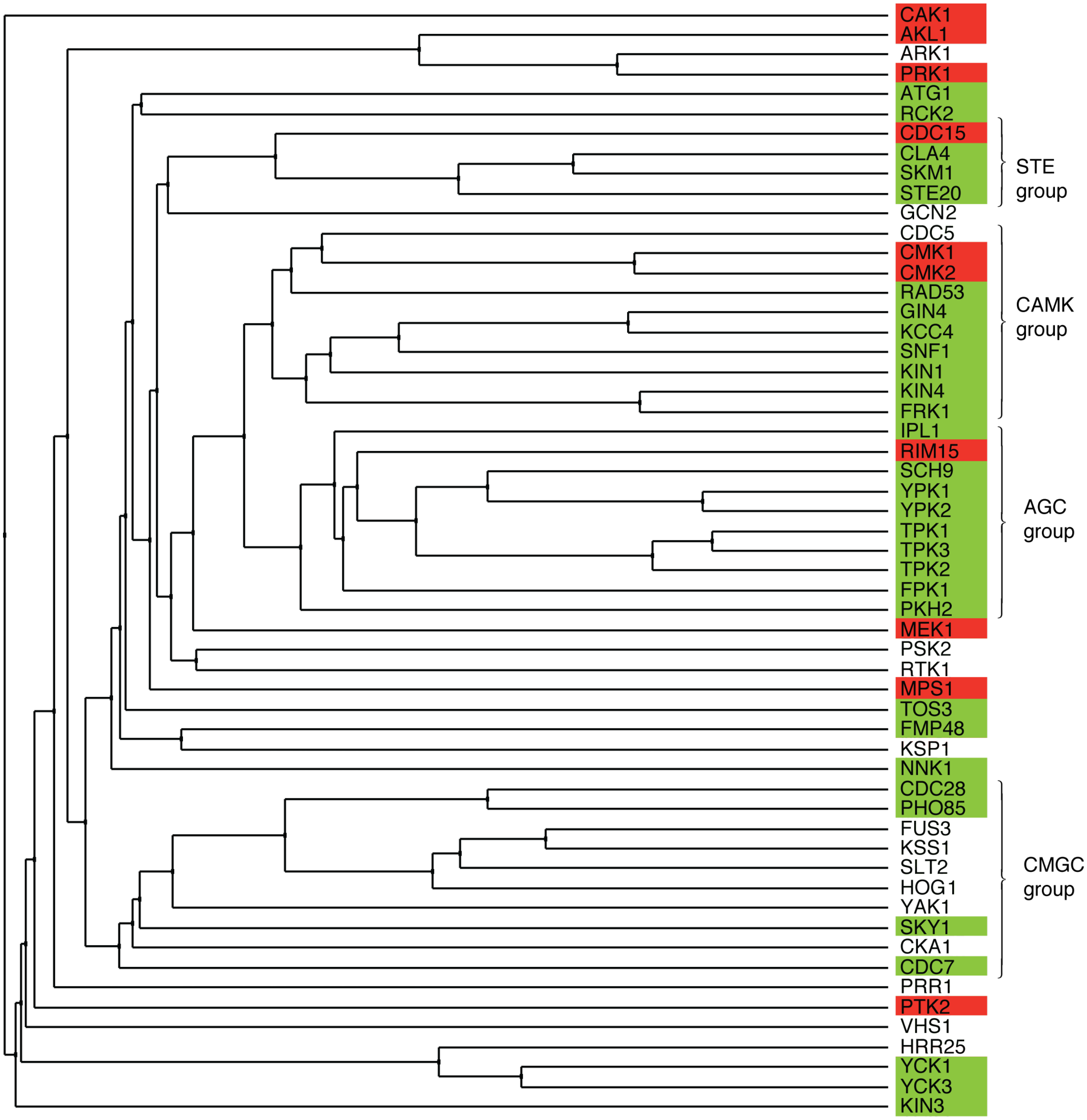

Figure S1. Dendrogram of yeast kinases of known phosphorylation site preference, related to Figure 1. The dendrogram was constructed for the 56 kinases listed in Table S1 using the program Jalview 2 (Waterhouse et al., 2009) from a reported catalytic domain alignment (Mok et al., 2010). Ser- and Thr-selective kinases based on the criteria used in Table S1 are indicated in green and red, respectively. Kinase groups following the nomenclature of Hanks and Hunter (Hanks and Hunter, 1995) are shown.

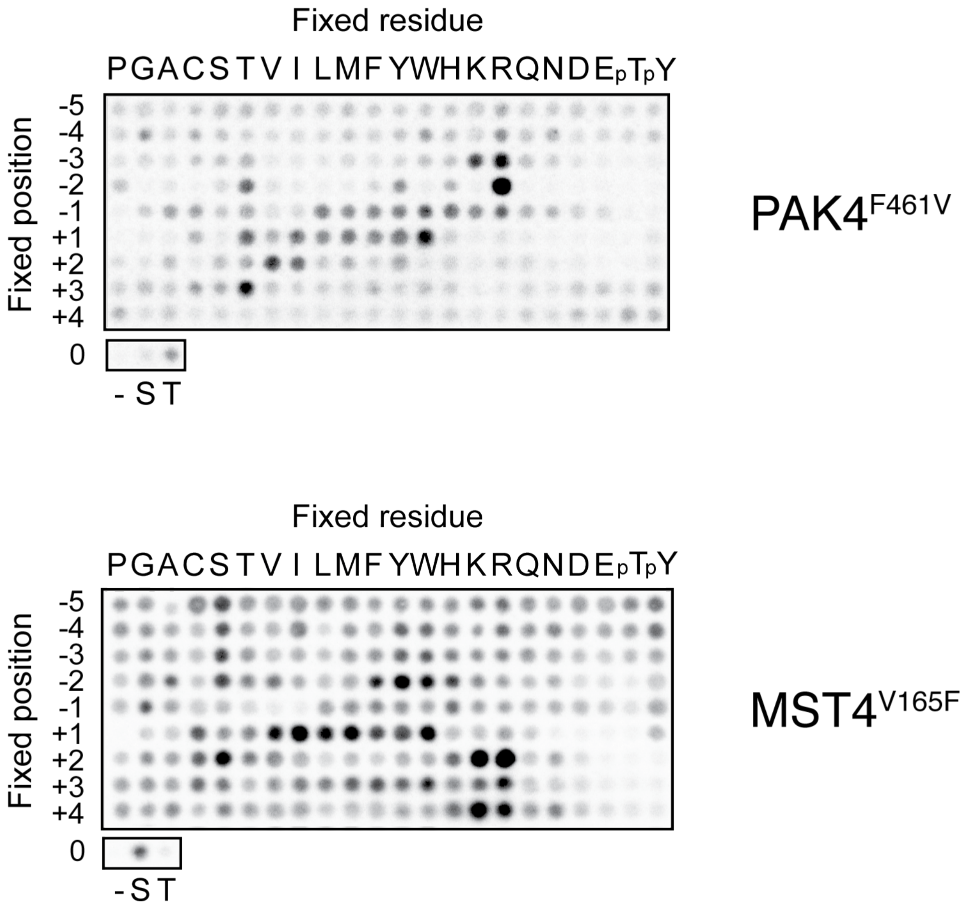

Figure S2. Peptide library analysis of PAK4 and MST4 mutant kinases, related to Table 1. The peptide substrate specificities of $PAK4^{F461V}$ and $MST4^{V165F}$ were determined using a positional scanning peptide library with the general sequence YAXXXXX-S/T-XXXXAGKK(biotin). Spot intensities reflect the extent of radiolabel incorporation from [γ-^{33}P]ATP into the peptide mixture with the indicated residue at the indicated position relative to the phosphorylation site. All residues strongly selected by the WT kinases (Miller et al., 2008; Rennefahrt et al., 2007) are also selected by the corresponding DFG+1 mutant. Note that peptides with fixed Ser or Thr residues at the X positions have two possible sites of phosphorylation, so that they often produce an artificially higher signal. This artifact also leads to differences in the peptide array profiles between the WT and DFG+1 mutant kinases in which signals for the Ser and Thr-containing peptides are exchanged.

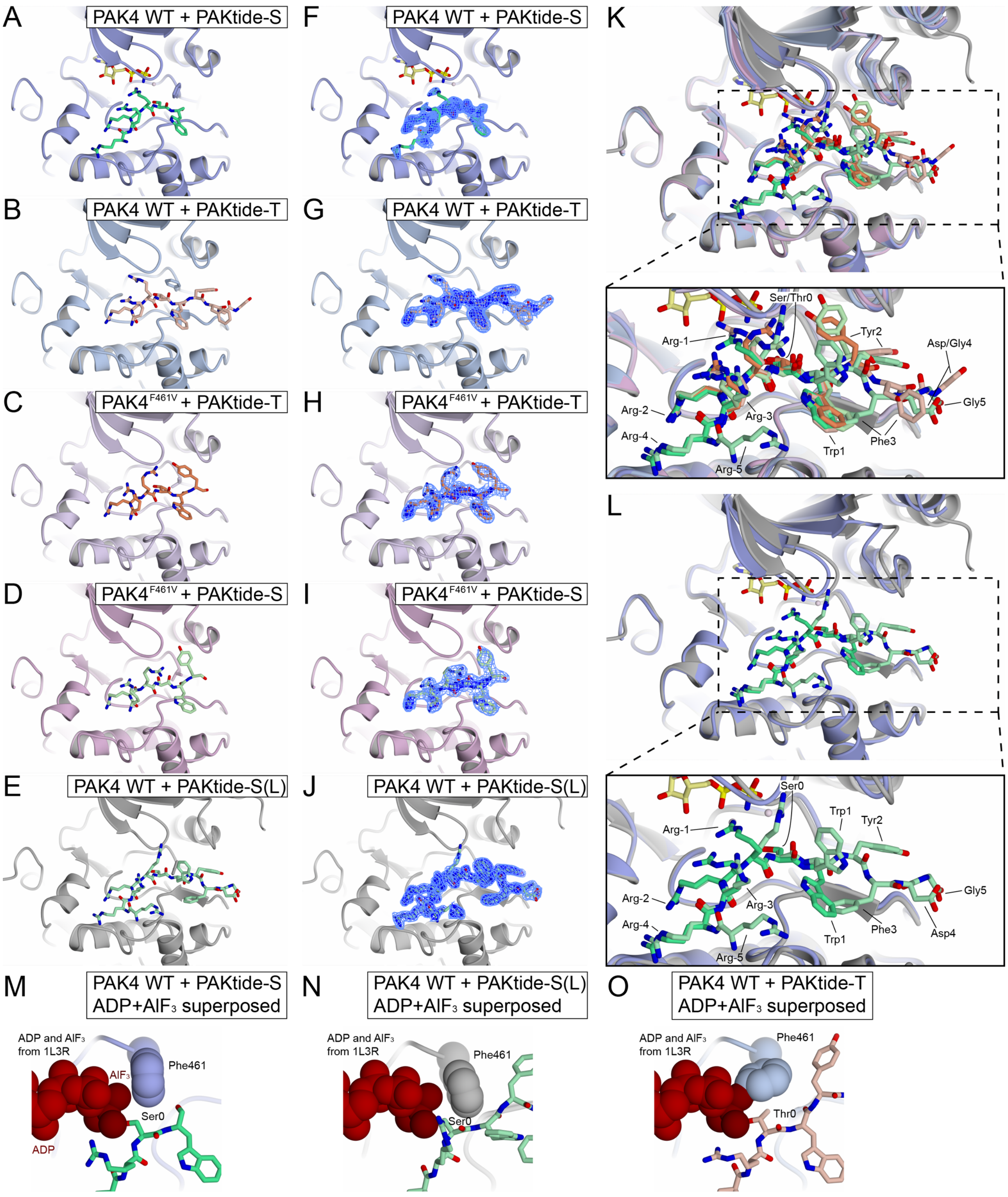

Figure S3. Detailed views of PAK4 and PAK4^{F461V} complexes with PAKtide-S and PAKtide-T, related to Figure 3. (**A-E**) Final refined models for each of the co-crystal structures as indicated. PAK4 is shown in cartoon format and PAKtide-S in stick format. AMP-PN is shown in stick format in panel A. (**F-J**) Same orientations as panels A-E, but with refined $2F_{obs}$-F_{calc} electron density shown. Two contour levels are shown, dark blue at 2σ and light blue at 1σ.

(**K**) Superposition of the refined structures shown in A-E. Peptide residues are labeled in the inset. (**L**) Superposition of the two PAK4 WT complexes with the two PAKtide-S peptides. Peptide residues are labeled in the inset. **(M-O)** Close-up views to illustrate the impact of Phe461 conformational change on the ATP binding site. To model the transition state we used the structure of PKA in complex with ADP and AlF_3 (PDB ID: 1L3R, Madhusudan *et al.*, 2002). The locations of ADP and AlF3 are shown as red or dark red spheres respectively. In the PAK4 WT + PAKtide-T structure Phe461 is expected to clash with the predicted location of the γ-phosphate of ATP.

Table S1. Phosphoacceptor preferences for 56 yeast kinases, related to Figure 1. The phosphorylation ratio of a pair of otherwise identical peptide mixtures having Ser or Thr as the phosphosite residue is shown from a published dataset (Mok et al., 2010). Because observed ratios appear to underestimate the extent of phosphorylation site discrimination (compare the S/T ratio for Snf1 with data from Figure 1B in the main text, for example) we chose a cutoff ratio of >1.6 to indicate a preference for Ser (indicated in green) or Thr (indicated in red) below. Kinase and ORF names are taken from the Saccharomyces Genome Database (Cherry et al., 2012), and kinase categories are from Hunter and Plowman (Hunter and Plowman, 1997).

Kinase	ORF	Kinase group	Family	S/T ratio	T/S ratio	Specificity (Ser or Thr)	DFG+1 residue
NNK1	YKL171W	Other	Unique	39.00	0.03	S	M
RCK2	YLR248W	CAMK	CAMK	39.00	0.03	S	L
ATG1	YGL180W	Other	ULK	10.35	0.10	S	F
KIN4	YOR233W	CAMK	AMPK	9.89	0.10	S	F
TPK2	YPL203W	AGC	PKA	7.80	0.13	S	F
YPK1	YKL126W	AGC	AKT	6.43	0.16	S	L
KIN1	YDR122W	CAMK	AMPK	5.61	0.18	S	L
SNF1	YDR477W	CAMK	AMPK	5.49	0.18	S	L
YPK2	YMR104C	AGC	AKT	5.43	0.18	S	L
FMP48	YGR052W	Other	KSP	5.31	0.19	S	H
TOS3	YGL179C	Other	ELM	5.20	0.19	S	V
SKM1	YOL113W	STE	STE20	4.76	0.21	S	F
PRR1	YKL116C	Other	Unique	4.71	0.21	S	L
STE20	YHL007C	STE	STE20	4.37	0.23	S	F
TPK1	YJL164C	AGC	PKA	4.33	0.23	S	F
SKY1	YMR216C	CMGC	CLK	4.18	0.24	S	N
TPK3	YKL166C	AGC	PKA	3.86	0.26	S	F
KCC4	YCL024W	CAMK	AMPK	3.40	0.29	S	M
CDC7	YDL017W	CMGC	CK2	3.07	0.33	S	L
IPL1	YPL209C	AGC	AUR	2.94	0.34	S	W
FRK1	YPL141C	CAMK	AMPK	2.92	0.34	S	F
CLA4	YNL298W	STE	STE20	2.86	0.35	S	F
PKH2	YOL100W	AGC	PDK1	2.61	0.38	S	T
KIN3	YAR018C	STE	NEK	2.45	0.41	S	L
SCH9	YHR205W	AGC	AKT	2.45	0.41	S	L
RAD53	YPL153C	CAMK	CHK2	2.28	0.44	S	L
YCK1	YHR135C	CK1	CK1	2.28	0.44	S	M
YCK3	YER123W	CK1	CK1	2.05	0.49	S	M
FPK1	YNR047W	AGC	S6K	2.00	0.50	S	L
CDC28	YBR160W	CMGC	CDK	1.95	0.51	S	L
PHO85	YPL031C	CMGC	CDK	1.94	0.51	S	L
GIN4	YDR507C	CAMK	AMPK	1.93	0.52	S	M
PSK2	YOL045W	CAMK	PIM	1.52	0.66	-	S
SLT2	YHR030C	CMGC	MAPK	1.46	0.69	-	L
CKA1	YIL035C	CMGC	CK2	1.37	0.73	-	L
HRR25	YPL204W	CK1	CK1	1.26	0.79	-	L
VHS1	YDR247W	Other	RAN	1.25	0.80	-	L
YAK1	YJL141C	CMGC	CLK	1.21	0.82	-	S
RTK1	YDL025C	YEAST	NPR	1.19	0.84	-	S
CDC5	YMR001C	Other	PLK	1.16	0.86	-	L
KSS1	YGR040W	CMGC	MAPK	1.14	0.88	-	L
KSP1	YHR082C	Other	KSP	1.10	0.91	-	L
ARK1	YNL020C	Other	PRK1	0.96	1.05	-	S
GCN2	YDR283C	Other	EIF2K	0.92	1.08	-	L
FUS3	YBL016W	CMGC	MAPK	0.88	1.14	-	L
HOG1	YLR113W	CMGC	MAPK	0.67	1.50	-	L
MEK1	YOR351C	CAMK	Unique	0.56	1.78	T	I
MPS1	YDL028C	Other	TTK	0.55	1.83	T	I
RIM15	YMR139W	AGC	RIM15	0.54	1.87	T	L
CMK1	YFR014C	CAMK	CAMK	0.48	2.10	T	I
PTK2	YJR059W	YEAST	PTK	0.46	2.19	T	I
CMK2	YOL016C	CAMK	CAMK	0.45	2.21	T	I
AKL1	YBR059C	Other	PRK1	0.42	2.37	T	S
CAK1	YFL029C	CMGC	CDK	0.42	2.40	T	I
PRK1	YIL095W	Other	PRK1	0.32	3.12	T	S
CDC15	YAR019C	STE	STE20	0.21	4.70	T	V

Table S2. Phosphoacceptor specificity of selected human kinases, related to Figure 1.

Kinase	Specificity (Reference)	DFG+1 residue
PKC-α	Ser (Ferrari et al., 1985; House et al., 1987)	Met
PKC-β	Ser (Ferrari et al., 1985; House et al., 1987)	Met
RSK1	Ser (Leighton et al., 1995)	Phe
LKB1	Thr (Lizcano et al., 2004)	Val
LRRK2	Thr (Nichols et al., 2009)	Ile
PDK1	Thr (Mora et al., 2004)	Thr
TTK1	Thr (Lindberg et al., 1993)	Ile

Table S3. Rates of reaction for kinase peptide phosphorylation assays, related to Figure 1. Rates show the mean values for data shown in Figure 1 in the main text.

Kinase	Peptide	Phosphorylation rate, μM/min/μg kinase
WT PKA	GGRRRRRSWYFGGGK	280
	GGRRRRRTWYFGGGK	14
PKA^{F187V}	GGRRRRRSWYFGGGK	20
	GGRRRRRTWYFGGGK	97
WT Snf1	ALARAASAAALAKKK	7.5
	ALARAATAAALAKKK	0.28
$Snf1^{L198S}$	ALARAASAAALAKKK	0.053
	ALARAATAAALAKKK	0.40
$Snf1^{L198V}$	ALARAASAAALAKKK	0.13
	ALARAATAAALAKKK	0.79
$Snf1^{L198I}$	ALARAASAAALAKKK	0.10
	ALARAATAAALAKKK	0.46
$Snf1^{L198M}$	ALARAASAAALAKKK	1.2
	ALARAATAAALAKKK	1.5
$Snf1^{L198F}$	ALARAASAAALAKKK	3.5
	ALARAATAAALAKKK	0.40
ROCK1	ARKRERAYSFGHHA	0.084
	ARKRERAYTFGHHA	0.16

SUPPLEMENTAL EXPERIMENTAL PROCEDURES

Plasmids and mutagenesis

The bacterial expression construct for the catalytic domain of human PAK4 (UniProt ID O96013) in a modified pET28 vector with an N-terminal hexa-histidine (6xHis) tag removable by TEV protease, was previously described (Ha et al., 2012). The catalytic domain of human MST4 (UniProt ID Q9P289) encoding residues 2-300 was subcloned into pCDF-Duet (Novagen) using EcoRI and HindIII and expressed with an N-terminal 6xHis tag removable by TEV protease. Mammalian expression vectors for GFP-PAK4 catalytic domain and GST-BAD (pEBG-BAD, mouse) were previously described (Ha et al., 2012; Tan et al., 2000). The bacterial expression vector for the 6xHis tagged mouse PKA catalytic domain (pET15b PKA cat) generated by Susan Taylor's laboratory (Narayana et al., 1997) was obtained from Addgene. The bacterial expression vector to produce the yeast Snf1 catalytic domain as an N-terminal GST fusion protein (pGEX4T-Snf1 cat) was previously described (Lee et al., 2012).

Point mutants were prepared using the QuikChange protocol (Stratagene) using the following primer pairs:

Gene	Mutant	Primer pair
PAK4	F461V	5' TGTCAGACTTTGGGGTCTGCGCCCAGGTG 3'<br>5' CACCTGGGCGCAGACCCCAAAGTCTGACA 3'
MST4	V165F	5' GCTGATTTTGGATTTGCTGGTCAGCTG 3'<br>5' AGCTGACCAGCAAATCCAAAATCAGC 3'
PKA	F187V	5' GTGACAGACTTCGGTGTTGCCAAGCGTGTG 3'<br>5' CACACGCTTGGCAACACCGAAGTCTGTCAC 3'
SNF1	L198S	5' GTAAAGATTGCCGATTTTGGTAGCTCAAACATCATGACTGATGGT 3'<br>5' ACCATCAGTCATGATGTTTGAGCTACCAAAATCGGCAATCTTTAC 3'
SNF1	L198V	5' GTAAAGATTGCCGATTTTGGTGTGTCAAACATCATGACTGATGGT 3'<br>5' ACCATCAGTCATGATGTTTGACACACCAAAATCGGCAATCTTTAC 3'
SNF1	L198I	5' GTAAAGATTGCCGATTTTGGTATTTCAAACATCATGACTGATGGT 3'<br>5' ACCATCAGTCATGATGTTTGAAATACCAAAATCGGCAATCTTTAC 3'
SNF1	L198M	5' GTAAAGATTGCCGATTTTGGTATGTCAAACATCATGACTGATGGT 3'<br>5' ACCATCAGTCATGATGTTTGACATACCAAAATCGGCAATCTTTAC 3'
SNF1	L198F	5' GTAAAGATTGCCGATTTTGGTTTTTCAAACATCATGACTGATGGT 3'<br>5' ACCATCAGTCATGATGTTTGAAAAACCAAAATCGGCAATCTTTAC 3'
BAD	S112T	5' CGGAGTCGCCACAGTACGTACCCAGCCGGGACC 3'<br>5' GGTCCCGGCTGGGTACGTACTGTGGCGACTCCG 3'

Protein expression and purification

The recombinant catalytic domains of WT PAK4 and PAK4^{F461V} were expressed as an N-terminal 6xHis fusion proteins in BL21-CodonPlus(DE3)RILP cells, and induced with 0.5 mM IPTG for 24 hours at 18°C. The harvested pellets were suspended in lysis buffer (20 mM Tris-HCl pH 8.0, 100 mM NaCl, 1 mM tris (2-carboxyethyl)phosphine (TCEP), and 0.1 mM PMSF) and lysed by sonication. The supernatants were affinity purified by HisTrap chelating column (GE) and then resolved over Resource Q (GE) and Superdex 75 10/300GL (GE Healthcare) columns. Purified proteins were concentrated to 5 mg/ml in 20 mM Tris-HCl pH 8.0, 150 mM NaCl, and 1 mM DTT with 1 mM phosphoaminophosphonic acid-adenylate ester (AMP-PNP) and 5 mM $MgCl_2$ for crystallization trials. For the PAK4 + PAKtide-S(L) complex, PAK4 was overexpressed and purified as described before (Eswaran et al., 2007).

Recombinant N-terminally 6xHis-tagged MST4 catalytic domain (WT and V165F mutant) was purified from an *E. coli* expression system. Following incubation to OD_{600} of 0.6 overnight protein expression was induced at 16 °C by addition of 3 mM IPTG. Cells were pelleted and resuspended in 20 mM Tris-HCl pH 8.0, 500 mM NaCl, 20 mM imidazole, 10% glycerol, 100 µg/ml lysozyme and 10 µg/ml DNase I. Cells were lysed by three cycles of freeze/thaw, followed by sonication, and the lysate was clarified by centrifugation at 20,000 rpm for 1 hour at 4 °C. Affinity purification of 6xHis-MST4 was achieved by flowing the supernatant over a HisTrap (GE) nickel column. Following column washing, MST4 was eluted with 400 mM imidazole and the 6xHis tag removed by overnight incubation with TEV protease and dialysis against 20 mM Tris pH 8.0, 500 mM NaCl, 20 mM imidazole, 10% glycerol and 1 mM DTT. Following cleavage and dialysis the sample was applied to a second HisTrap column to remove uncleaved protein. Final purification was by FPLC gel filtration using an S200 preparative column (GE).

6xHis tagged PKA catalytic domain (WT and mutant) expression was induced at 16 ºC for 18 hours with 0.4 mM IPTG in BL21(DE3) *E. coli* cells grown in terrific broth. Bacterial pellets were suspended in 20 mM Tris-HCl, pH 7.5, 140 mM NaCl, 3 mM β-mercaptoethanol, 10 µg/ml leupeptin, 10 µg/ml pepstatin A, and lysed by incubation with 0.2 mg/ml lysozyme, 0.4% Igepal CA630, 1 mM PMSF, 0.03 U/µl DNase I and 13 mM $MgCl_2$ with rotating for 30 min at 4 ºC. The lysate was centrifuged and supernatants were incubated in batch with Talon beads (Clontech, 0.5 ml per 200 ml culture volume) with rotating at 4 ºC for 1 hour. Beads were pelleted, suspended in PBS containing 0.5% Igepal CA630 and loaded into a column. The resin was washed with PBS/Igepal CA630 followed by 20 mM Tris-HCl, pH 7.5, 500 mM NaCl and 10 mM imidazole. Protein was eluted from the column with 20 mM Tris-HCl, pH 7.5, 100 mM NaCl, 250 mM imidazole, 1 mM DTT, 10 µg/ml leupeptin, and then dialyzed overnight into 10 mM HEPES, pH 7.4, 100 mM NaCl, 1 mM DTT, 10% glycerol.

GST-Snf1 catalytic domain and its point mutants were produced in E. coli, purified by one-step affinity purification on glutathione-Sepharose 4B (GE Healthcare), and activated in vitro by phosphorylation with recombinant Elm1 kinase (obtained from Martin Schmidt, University of Pittsburgh) as previously described (Lee et al., 2012). Recombinant human ROCK1 (produced in Sf9 insect cells) was purchased from R&D Systems (catalog number 4590-KS).

Synthetic peptides

ROCK substrate peptides (ARKRERAYSFGHHA and ARKRERAYTFGHHA) and the short version of PAKtide-S used for crystallography (RRRRSWY) were synthesized and HPLC purified by the Tufts University Core Facility (Boston, MA). The sequence of the long version of PAKtide-S, PAKtide-S(L) used for crystallography was RRRRRSWYFDG. PAK and PKA substrates (GGRRRRRSWYFGGGK and GGRRRRRTWYFGGGK), MST4 substrates (NKGYNSLRRKK and NKGYNTLRRKK) and Snf1 substrates (ALARAASAAALAKKK and ALARAATAAALAKKK) were prepared using standard Fmoc solid phase peptide synthesis procedures (Wellings and Atherton, 1997) on a Tetras peptide synthesizer (Creosalus). Peptides were synthesized on 150 mg Wang resin preloaded with Fmoc-Lys (Advanced ChemTech) using DMF (American Bioanalytical) as the solvent and HBTU/HOBt (Anaspec) and DIPEA (Sigma-Aldrich) as coupling reagents. Fmoc-protected amino acids were purchased from Advanced ChemTech or Anaspec. Peptides were cleaved from the resin in 3 ml reagent K (83%

trifluoroacetic acid, 2.5% triisopropylsilane, 2.5% 1,2-ethanedithiol, 4% thioanisole, 4% phenol and 4% H_2O). Peptides were precipitated in diethyl ether, recovered by centrifugation and dried under a stream of argon. Peptides were purified by reversed phase HPLC on a C18 column using a gradient of acetonitrile in water with 0.1% TFA as the solvent, and lyophilized to dryness. Peptide identity was verified by MALDI-TOF mass spectrometry. For peptides containing Tyr or Trp residues, peptide stock solutions were quantified spectrophotometrically at 280 nm. Otherwise, peptides were quantified by fluorescamine assay (Udenfriend et al., 1972) using a peptide of known concentration as a standard.

Kinase assays

PAK assays were performed in duplicate using 100 nM WT PAK4 or 500 nM $PAK4^{F461V}$ catalytic domains with peptide concentrations in two-fold increments ranging from 0.625 μM to 640 μM in PAK4 kinase buffer (50 mM HEPES, pH 7.5, 12.5 mM NaCl, 10 mM $MgCl_2$, 1 mM $MnCl_2$, 1mM DTT). MST4 kinase assays were performed similarly in duplicate using 0.25 nM WT MST4 or 500 nM $MST4^{V165F}$ catalytic domains with a range of 0.625 μM to 1 mM peptide in MST4 kinase buffer (20 mM HEPES, pH 7.5, 10 mM $MgCl_2$, 1mM DTT). Reactions were initiated by adding ATP to a final concentration of 100 μM with 0.25 μCi/μl [γ-^{33}P]ATP (Perkin-Elmer) and incubated at 30°C for 10 min. Aliquots were spotted onto P81 phosphocellulose filters, which were quenched and processed as described in the main text.

PKA assays were done similarly as above in triplicate with a single concentration of peptide (10 μM) and kinase (20 ng/ml), using 50 mM HEPES, pH 7.4, 100 mM KCl, 10 mM $MgCl_2$, 1 mM DTT as the reaction buffer and 50 μM ATP (0.1 μCi/μl [γ-^{33}P]ATP), with time points taken at 3, 6 and 9 min. ROCK1 assays were done in duplicate at 5 μM peptide and 25 nM ROCK1 and 25 mM HEPES, pH 7.4, 12.5 mM β-glycerophosphate, 25 mM $MgCl_2$, 5 mM EGTA, 2 mM EDTA, 0.25 mM DTT as the reaction buffer and 50 μM ATP (0.1 μCi/μl [γ-^{33}P]ATP), and time points were taken at 5, 10 and 15 min. Snf1 assays were done at 40 μM peptide and 250 nM kinase in 50 mM HEPES, pH 7.4, 10 mM $MgCl_2$, 50 mM NaCl, 0.5 mM DTT with 100 μM ATP (0.05 μCi/μl [γ-^{33}P]ATP), with time points taken at 5, 10 and 15 min. Reaction rates for all peptide kinase assays were linear at all substrate concentrations used.

Peptide library analysis

The peptide substrate specificities of $PAK4^{F461V}$ and $MST4^{V165F}$ were determined using a positional scanning peptide library as previously described (Mok et al., 2010). The library consisted of 200 peptide mixtures with the general sequence YAXXXXXZXXXXAGKK(biotin). For 198 of the mixtures, one of the nine X positions was equal to one of the 20 unmodified amino acids, phosphothreonine or phosphotyrosine, the remaining X positions were an equimolar mixture of the 17 amino acids excluding Cys, Ser and Thr, and Z was equal to an even mixture of Ser and Thr. The two remaining peptides had all X positions as mixtures with Z equal to either Ser or Thr to provide phosphorylation site preference. Peptide mixtures (50 μM) were incubated for 2 hr at 30 ºC with kinase in the corresponding reaction buffer indicated above and 50 μM ATP with 0.03 μCi/μl [γ-^{33}P]ATP in 2 μl reactions in a 1536-well plate. Aliquots (200 nl) of each reaction were transferred to a streptavidin membrane (Promega SAM2 biotin capture membrane), which was quenched and washed as described (Mok et al., 2010). Membranes were air-dried and exposed to a phosphor screen to visualize radiolabel incorporation into peptides.

Protein crystallization and data collection

To obtain WT PAK4 and PAK4^{F461V} catalytic domain crystals with both PAKtide-T (GGRRRRRTWYFGGGK) and PAKtide-S (RRRRSWY) we used the vapor diffusion hanging drop method. For co-crystallization of the PAK4^{F461V} with PAKtide-T or -S peptide we pre-incubated PAK4^{F461V} with peptides in a 1:1.3 ratio and grew the PAK4^{F461V} co-crystals with petptide by mixing this in a 1:1 volume ratio with precipitant solution containing 0.1 M Tris-HCl pH 7.5, 1.5 – 2.0 M NaOAc at room temperature. For the WT PAK4 with PAKtide-T or -S peptide we soaked WT PAK4 crystals, grown against 0.1 M Tris-HCl pH 7.5, 1.5 – 2.0 M NaOAc at room temperature, by adding 1 mM of peptides to the crystallization drop. WT PAK4 crystals were then dehydrated for 8 – 15 hours dehydration in 0.1 M Tris-HCl pH 7.5 and 2.4 M NaOAc reservoir solution and the presence of excess PAKtide-T or -S. Prior to flash freezing, these crystals were equilibrated over 2.4 M NaOAc as a cryoprotectant. WT PAK4 + PAKtide-S data were collected to 1.85 Å resolution at the Advanced Photon Source (APS) beamline 24-ID-E and WT PAK4 + PAKtide-T data were collected to 2.0 Å at the National Synchrotron Light Source (NSLS) beamline X25. PAK4^{F461V} + PAKtide-T and PAK4^{F461V} + PAKtide-S data were collected to 2.3 Å and 2.4 Å at the National Synchrotron Light Source (NSLS) beamline X25 respectively. To obtain crystals of PAK4 in complex with PAKtide-S(L) (RRRRRSWYFDG), PAK4 was concentrated to 9.3 mg/ml, and the complex was crystallized in 96 well sitting drop Greiner plates using the vapor diffusion method at 4°C, mixing 100 nl protein solution with a well solution containing 1.7 M ammonium sulfate, 15% PEG400, 0.1 M Tris pH 8.0. Crystals were frozen in the crystallization buffer containing 20% ethylene glycol. Data were collected at the Swiss light source beamline SLS-X10 at a wavelength of 0.97910 Å.

Structure determination and refinement

Crystallographic data were processed using the HKL2000 package (Otwinowski and Minor, 1997). We generated initial phases by molecular replacement using the program Phaser (McCoy, 2007) and the previously determined crystal structure of PAK4 catalytic domain with a pseudosubstrate inhibitor peptide (PDB ID: 4FIF) (Ha et al., 2012) as the search model. This yielded Translation Factor Z-scores (TFZ) of 45.6, 49.3, 46.7, and 48.3 for the WT PAK4 + PAKtide-T, WT PAK4 + PAKtide-S, PAK4^{F461V} + PAKtide-T, and PAK4^{F461V} + PAKtide-S co-crystal structures respectively. Refinements were conducted using Refmac5 (Murshudov et al., 2011) with a maximum-likelihood target, TLS (translation, libration, screw), and medium non-crystallographic symmetry restraints. Model building was conducted in COOT (Emsley et al., 2010) and model validation using MolProbity (Chen et al., 2010) with all built residues within favored or allowed Ramachandran regions. Good electron density is observed for each of the kinase domains and their bound peptides, although bound nucleotide could only be built for the PAK4 + PAKtide-S structure. We initially built AMP-PNP into the PAK4 + PAKtide-S structure, however, recent work has suggested that hydrolysis of AMP-PNP can occur in crystal (Bastidas et al., 2013). We therefore modeled AMP-PN and Mg^{2+} into the structure instead of AMP-PNP and observed a drop in R-free (from 23.6% to 23.3%) and a drop in R-factor (from 19.4% to 19.2%). AMP-PN also provided a better fit of the model to the electron density. We concluded that hydrolysis of AMP-PNP had occurred in this case and built the model for WT PAK4 + PAKtide-S to include AMP-PN and Mg^{2+}.

SUPPLEMENTAL REFERENCES

Bastidas, A.C., Deal, M.S., Steichen, J.M., Guo, Y., Wu, J., and Taylor, S.S. (2013). Phosphoryl transfer by protein kinase A is captured in a crystal lattice. J. Am. Chem. Soc. *135*, 4788-98.

Chen, V.B., Arendall, W.B., 3rd, Headd, J.J., Keedy, D.A., Immormino, R.M., Kapral, G.J., Murray, L.W., Richardson, J.S., and Richardson, D.C. (2010). MolProbity: all-atom structure validation for macromolecular crystallography. Acta Crystallogr. D Biol. Crystallogr. *66*, 12-21.

Cherry, J.M., Hong, E.L., Amundsen, C., Balakrishnan, R., Binkley, G., Chan, E.T., Christie, K.R., Costanzo, M.C., Dwight, S.S., Engel, S.R.*, et al.* (2012). Saccharomyces Genome Database: the genomics resource of budding yeast. Nucleic Acids Res. *40*, D700-705.

Emsley, P., Lohkamp, B., Scott, W.G., and Cowtan, K. (2010). Features and development of Coot. Acta Crystallogr. D Biol. Crystallogr. *66*, 486-501.

Eswaran, J., Lee, W.H., Debreczeni, J.E., Filippakopoulos, P., Turnbull, A., Fedorov, O., Deacon, S.W., Peterson, J.R., and Knapp, S. (2007). Crystal Structures of the p21-activated kinases PAK4, PAK5, and PAK6 reveal catalytic domain plasticity of active group II PAKs. Structure *15*, 201-213.

Ferrari, S., Marchiori, F., Borin, G., and Pinna, L.A. (1985). Distinct structural requirements of Ca2+/phospholipid-dependent protein kinase (protein kinase C) and cAMP-dependent protein kinase as evidenced by synthetic peptide substrates. FEBS Lett. *184*, 72-77.

Ha, B.H., Davis, M.J., Chen, C., Lou, H.J., Gao, J., Zhang, R., Krauthammer, M., Halaban, R., Schlessinger, J., Turk, B.E.*, et al.* (2012). Type II p21-activated kinases (PAKs) are regulated by an autoinhibitory pseudosubstrate. Proc. Natl. Acad. Sci. USA *109*, 16107-16112.

Hanks, S.K., and Hunter, T. (1995). Protein kinases 6. The eukaryotic protein kinase superfamily: kinase (catalytic) domain structure and classification. FASEB J. *9*, 576-596.

House, C., Wettenhall, R.E., and Kemp, B.E. (1987). The influence of basic residues on the substrate specificity of protein kinase C. J. Biol. Chem. *262*, 772-777.

Hunter, T., and Plowman, G.D. (1997). The protein kinases of budding yeast: six score and more. Trends Biochem. Sci. *22*, 18-22.

Lee, Y.J., Jeschke, G.R., Roelants, F.M., Thorner, J., and Turk, B.E. (2012). Reciprocal phosphorylation of yeast glycerol-3-phosphate dehydrogenases in adaptation to distinct types of stress. Mol. Cell. Biol. *32*, 4705-4717.

Leighton, I.A., Dalby, K.N., Caudwell, F.B., Cohen, P.T., and Cohen, P. (1995). Comparison of the specificities of p70 S6 kinase and MAPKAP kinase-1 identifies a relatively specific substrate for p70 S6 kinase: the N-terminal kinase domain of MAPKAP kinase-1 is essential for peptide phosphorylation. FEBS Lett. *375*, 289-293.

Lindberg, R.A., Fischer, W.H., and Hunter, T. (1993). Characterization of a human protein threonine kinase isolated by screening an expression library with antibodies to phosphotyrosine. Oncogene *8*, 351-359.

Lizcano, J.M., Goransson, O., Toth, R., Deak, M., Morrice, N.A., Boudeau, J., Hawley, S.A., Udd, L., Makela, T.P., Hardie, D.G., *et al.* (2004). LKB1 is a master kinase that activates 13 kinases of the AMPK subfamily, including MARK/PAR-1. EMBO J. *23*, 833-843.

Madhusudan, Akamine, P., Xuong, N.H., Taylor, S.S. (2002). Crystal structure of a transition state mimic of the catalytic subunit of cAMP-dependent protein kinase. Nat. Struct. Biol. *9*, 273-7.

McCoy, A.J. (2007). Solving structures of protein complexes by molecular replacement with Phaser. Acta Crystallogr. D Biol. Crystallogr. *63*, 32-41.

Miller, M.L., Jensen, L.J., Diella, F., Jorgensen, C., Tinti, M., Li, L., Hsiung, M., Parker, S.A., Bordeaux, J., Sicheritz-Ponten, T., *et al.* (2008). Linear motif atlas for phosphorylation-dependent signaling. Sci. Signal. *1*, ra2.

Mok, J., Kim, P.M., Lam, H.Y., Piccirillo, S., Zhou, X., Jeschke, G.R., Sheridan, D.L., Parker, S.A., Desai, V., Jwa, M., *et al.* (2010). Deciphering protein kinase specificity through large-scale analysis of yeast phosphorylation site motifs. Sci. Signal. *3*, ra12.

Mora, A., Komander, D., van Aalten, D.M., and Alessi, D.R. (2004). PDK1, the master regulator of AGC kinase signal transduction. Semin. Cell Dev. Biol. *15*, 161-170.

Murshudov, G.N., Skubak, P., Lebedev, A.A., Pannu, N.S., Steiner, R.A., Nicholls, R.A., Winn, M.D., Long, F., and Vagin, A.A. (2011). REFMAC5 for the refinement of macromolecular crystal structures. Acta Crystallogr. D Biol. Crystallogr. *67*, 355-367.

Narayana, N., Cox, S., Nguyen-huu, X., Ten Eyck, L.F., and Taylor, S.S. (1997). A binary complex of the catalytic subunit of cAMP-dependent protein kinase and adenosine further defines conformational flexibility. Structure *5*, 921-935.

Nichols, R.J., Dzamko, N., Hutti, J.E., Cantley, L.C., Deak, M., Moran, J., Bamborough, P., Reith, A.D., and Alessi, D.R. (2009). Substrate specificity and inhibitors of LRRK2, a protein kinase mutated in Parkinson's disease. Biochem. J. *424*, 47-60.

Otwinowski, Z., and Minor, W. (1997). Processing of X-ray diffraction data collected in oscillation mode. Methods Enzymol. *276*, 307-326.

Rennefahrt, U.E., Deacon, S.W., Parker, S.A., Devarajan, K., Beeser, A., Chernoff, J., Knapp, S., Turk, B.E., and Peterson, J.R. (2007). Specificity profiling of Pak kinases allows identification of novel phosphorylation sites. J. Biol. Chem. *282*, 15667-15678.

Tan, Y., Demeter, M.R., Ruan, H., and Comb, M.J. (2000). BAD Ser-155 phosphorylation regulates BAD/Bcl-XL interaction and cell survival. J. Biol. Chem. *275*, 25865-25869.

Udenfriend, S., Stein, S., Bohlen, P., Dairman, W., Leimgruber, W., and Weigele, M. (1972). Fluorescamine: a reagent for assay of amino acids, peptides, proteins, and primary amines in the picomole range. Science *178*, 871-872.

Waterhouse, A.M., Procter, J.B., Martin, D.M., Clamp, M., and Barton, G.J. (2009). Jalview Version 2--a multiple sequence alignment editor and analysis workbench. Bioinformatics *25*, 1189-1191.

Wellings, D.A., and Atherton, E. (1997). Standard Fmoc protocols. Methods Enzymol. *289*, 44-67.
